# A Computational Framework for the Design and Mechanical Assessment of Biodegradable Airway Stents: Interaction with Rabbit Tracheal Tissue and Preliminary In Vivo Observations

**DOI:** 10.3390/jfb17080419

**Published:** 2026-08-20

**Authors:** Ada Ayechu-Abendaño, Letizia Cella, Carmen Sánchez-González, Carmen Sánchez-Matás, José Luis López-Villalobos, Cristina Díaz-Jiménez, Rocío Fernández-Parra, Mauro Malvè

**Affiliations:** 1 Department of Engineering, Public University of Navarra (UPNA), Campus Arrosadía s/n, 31006 Pamplona, Spain; 2 Mechanical Systems Engineering, Empa—Swiss Federal Laboratories for Materials Science and Technology, Überlandstrasse 129, 8600 Dübendorf, Switzerland; 3 Doctoral School, Catholic University of Valencia San Vicente Mártir, San Agustín Square, 3, 46002 Valencia, Spain; 4 Veterinary Referral Hospital UCV, Catholic University of Valencia San Vicente Mártir, Avenida Pérez Galdós Street 51, 46018 Valencia, Spain; rocio.f.parra@ucv.es; 5 AIN—Asociación de la Industria Navarra, Ctra. Pamplona, 1. Edif. AIN, 31191 Cordovilla, Spain; cdiaz@ain.es; 6 Department of Thoracic Surgery, University Hospital Virgen de la Arrixaca, Ctra. Madrid-Cartagena, s/n, 30120 El Palmar, Spain; 7 Department of Thoracic Surgery, University Hospital Virgen del Rocío, Avenida Manuel Siurot s/n, 41013 Sevilla, Spain; 8 Department of Small Animal Medicine and Surgery, Faculty of Veterinary Medicine and Experimental Sciences, Catholic University of Valencia San Vicente Mártir, Quevedo Street 2, 46001 Valencia, Spain; 9 CIBER-BBN, Research Networking in Bioengineering, Biomaterials & Nanomedicine, Av. Monforte de Lemos, 3-5, Pabellón 11, Planta 0, 28029 Madrid, Spain; 10 Instituto de Materiales Avanzados y Matemáticas (INAMAT2), Public University of Navarre (UPNA), Edificio Jerónimo de Ayanz, Campus de Arrosadía, 31006 Pamplona, Spain

**Keywords:** biodegradable stent, airway stent, finite element analysis, trachea, PLA/PCL, 70/30 wt.% blend, AZ31 magnesium alloy, stent–tissue interaction, additive manufacturing

## Abstract

Current airway stents, including silicone and metallic devices, remain associated with important complications such as migration, restenosis, mucus retention and the need for repeated interventions. Biodegradable stents offer a promising alternative by providing temporary mechanical support while avoiding the long-term presence of a permanent implant. However, the influence of stent geometry and material properties on their mechanical performance and interaction with airway tissue is still not fully understood. This study presents a computational framework integrating computer-aided design and finite element analysis to investigate the mechanical behaviour of biodegradable tracheobronchial stents. Two stent architectures (X-pattern and W-pattern) were analysed over a range of wire thicknesses using two biodegradable materials: a PLA/PCL; 70/30 wt.% blend and AZ31 magnesium alloy. Radial compression, diameter recovery after radial compression and stent–tissue interaction simulations were performed to evaluate the influence of geometry, material selection and design parameters on device performance. The results suggested that both stent geometry and material properties strongly influence the mechanical behaviour of biodegradable airway stents, although they affect different aspects of the stent–tissue interaction. The X-pattern consistently exhibited greater resistance to radial compression, lower elastic diameter recovery after radial compression and improved maintenance of the expanded lumen compared with the W-pattern. Material properties primarily affected the magnitude of the mechanical response, as further confirmed by the quantitative contact-pressure analysis, with AZ31 providing greater radial support, while the spatial distributions of stress and strain within the tracheal wall were mainly governed by the stent architecture. Based on the computational analyses, X-pattern stents manufactured from the PLA/PCL; 70/30 wt.% blend were selected for in vivo evaluation in a rabbit model. Endoscopic observations revealed tissue features that were qualitatively consistent with the mechanical patterns predicted by the numerical simulations, although no direct causal relationship can be established from the available observations. These findings support the ability of the proposed framework to represent the principal aspects of stent–tissue interaction. The proposed computational framework provides a practical tool for the rational design and mechanical assessment of biodegradable airway stents and may facilitate the future development of customised airway prostheses.

## 1. Introduction

The human airway plays a vital role in respiration, allowing the passage of air to and from the lungs. However, various pathological conditions, such as malignant tumours, benign strictures, post-intubation stenosis and tracheobronchomalacia, can lead to airway obstruction, severely impairing breathing and quality of life [1,2]. To manage these conditions, airway stents are commonly used as a last-resort intervention to restore airway patency. These prostheses, typically manufactured from silicone, metallic or hybrid materials, provide structural support to maintain airway patency. However, their clinical effectiveness remains variable and, in some cases, unpredictable.

Despite their benefits, airway stents can present challenges related to their interaction with tracheal tissue. The placement of a foreign device in the airway may trigger biological responses, including inflammation, granulation tissue formation and fibrosis, which can lead to restenosis or obstruction [1,3,4,5]. In addition, stent migration and mechanical irritation may cause discomfort and other complications requiring re-intervention, including stent removal or replacement [6,7]. The extent of these complications depends on multiple factors, including stent material, geometry and patient-specific anatomical and physiological conditions [8,9].

A thorough understanding of the interaction between airway stents and tracheal tissue is therefore essential for improving stent design, minimising adverse effects and enhancing clinical outcomes. In particular, biodegradable airway stents have emerged as a promising alternative because they provide temporary mechanical support while gradually degrading after fulfilling their function, thereby avoiding the long-term presence of a permanent implant and the need for device removal.

Several experimental and preclinical studies have demonstrated the feasibility of biodegradable airway stents and reported encouraging biological outcomes in both animal models and selected adult and paediatric patients [7,10,11,12]. Nevertheless, current biodegradable airway stents still present important limitations. As highlighted by Guibert et al. [13], although the concept is highly attractive for the treatment of temporary airway stenoses, a considerable proportion of patients required subsequent re-stenting because the mechanical support was not maintained for a sufficient period. Consequently, biodegradable airway stents cannot yet be considered a routine alternative to conventional silicone devices, and further optimisation of their radial mechanical performance, degradation behaviour and material selection remains necessary. Biodegradable materials used in airway stents are designed to maintain airway patency for a predefined period while gradually degrading through hydrolysis or enzymatic processes. Among the biodegradable polymers investigated for airway stents, poly(lactic acid) (PLA), poly(caprolactone) (PCL), poly(glycolic acid) (PGA) and poly(lactic-co-glycolic acid) (PLGA) have attracted considerable attention because of their biocompatibility, controllable degradation behaviour and suitability for additive manufacturing [14,15,16]. In addition, biodegradable stents can be engineered as drug-eluting devices capable of locally delivering anti-inflammatory or anti-fibrotic agents, further improving their therapeutic potential.

Despite these advantages, significant challenges remain in optimising the degradation rate, mechanical strength and biocompatibility of biodegradable airway stents. Current research therefore focuses not only on developing novel biomaterials but also on exploiting advanced manufacturing technologies, particularly additive manufacturing, to fabricate patient-specific devices with tailored mechanical performance [13,17,18,19,20,21,22].

Since no biodegradable airway stents are currently commercially available, a better understanding of the relationship between stent design, material properties and airway tissue response is required before these devices can be translated into routine clinical practice. In particular, the mechanical performance of the stent must be carefully balanced to provide sufficient radial support while avoiding excessive tissue loading that could promote adverse biological responses.

Computational modelling offers a valuable tool for investigating these interactions in a systematic and cost-effective manner. Numerical approaches allow the evaluation of multiple design configurations, materials and loading conditions that would be difficult to assess experimentally alone. Furthermore, the integration of computer-aided design, finite element analysis and additive manufacturing technologies creates new opportunities for the development of patient-specific biodegradable airway prostheses. In this work, a computational framework combining parametric stent design and three-dimensional finite element modelling of the rabbit trachea was developed to investigate the mechanical interaction between biodegradable airway stents and tracheal tissue. The rabbit airway model was based on previous studies [23,24,25] and incorporates the principal anatomical components of the tracheal wall, including the cartilaginous rings and the posterior muscular membrane.

Previous work by the authors investigated the influence of selected geometric parameters on the mechanical response of biodegradable airway stents [23]. Building upon these results, the present study shifts the focus from isolated parameter variations towards a broader assessment of stent architecture, material selection and tissue interaction, with the aim of understanding how these factors collectively influence radial support and local tissue loading.

Two parameterised stent architectures, denoted as X-pattern and W-pattern, were considered to evaluate the influence of geometric design on mechanical performance. In addition, two biodegradable materials with markedly different mechanical properties, namely poly(lactic acid)/poly(caprolactone) (PLA/PCL; 70/30 wt.%) and AZ31 magnesium alloy, were investigated over a range of wire thicknesses representative of additively manufactured devices.

The study combines radial compression simulations, hysteresis and diameter recovery analyses after radial compression, quantitative assessment of radial mechanical performance, and stent–tissue interaction simulations to characterise how geometry, material selection and design parameters affect both the mechanical behaviour of the device and the resulting loading conditions within the tracheal wall. Finally, preliminary in vivo observations obtained from rabbit implantation experiments are qualitatively compared with the computational predictions without attempting to establish a direct causal relationship in order to explore potential relationships between the predicted mechanical environment and the observed tissue response.

The overall objective of this work is therefore to establish a systematic framework for the design and evaluation of biodegradable airway stents, providing new insight into the role of stent architecture and material selection in determining radial mechanical performance, airway support and tissue interaction. Such knowledge is expected to contribute to the future development of customised biodegradable airway prostheses tailored to specific anatomical and clinical requirements.

## 2. Materialsand Methods

The geometrical models of the rabbit trachea and the airway stents were created in SolidWorks ver. 31.0 (Dassault Systèmes, Waltham, MA, USA) and directly linked to the finite element software ANSYS Release 2020 R2 (Ansys Inc., Canonsburg, PA, USA), where all numerical simulations were performed. Both the tracheal model and the stent geometries were fully parameterised, allowing automatic modification of their main geometric characteristics within the computer-aided design (CAD) environment while simultaneously updating the finite element model. This integrated modelling workflow provides a flexible computational framework for evaluating multiple stent configurations without manual remeshing or model reconstruction. The present study focuses on the influence of stent architecture, biodegradable material and wire thickness on both radial mechanical performance and stent–tissue interaction. Since the objective of this work was to investigate the initial mechanical interaction between the stent and the tracheal wall immediately after deployment, the biodegradable materials were therefore modelled using their initial constitutive properties. Time-dependent degradation and the associated reduction in mechanical stiffness were beyond the scope of the present computational framework.

**Figure 1:**
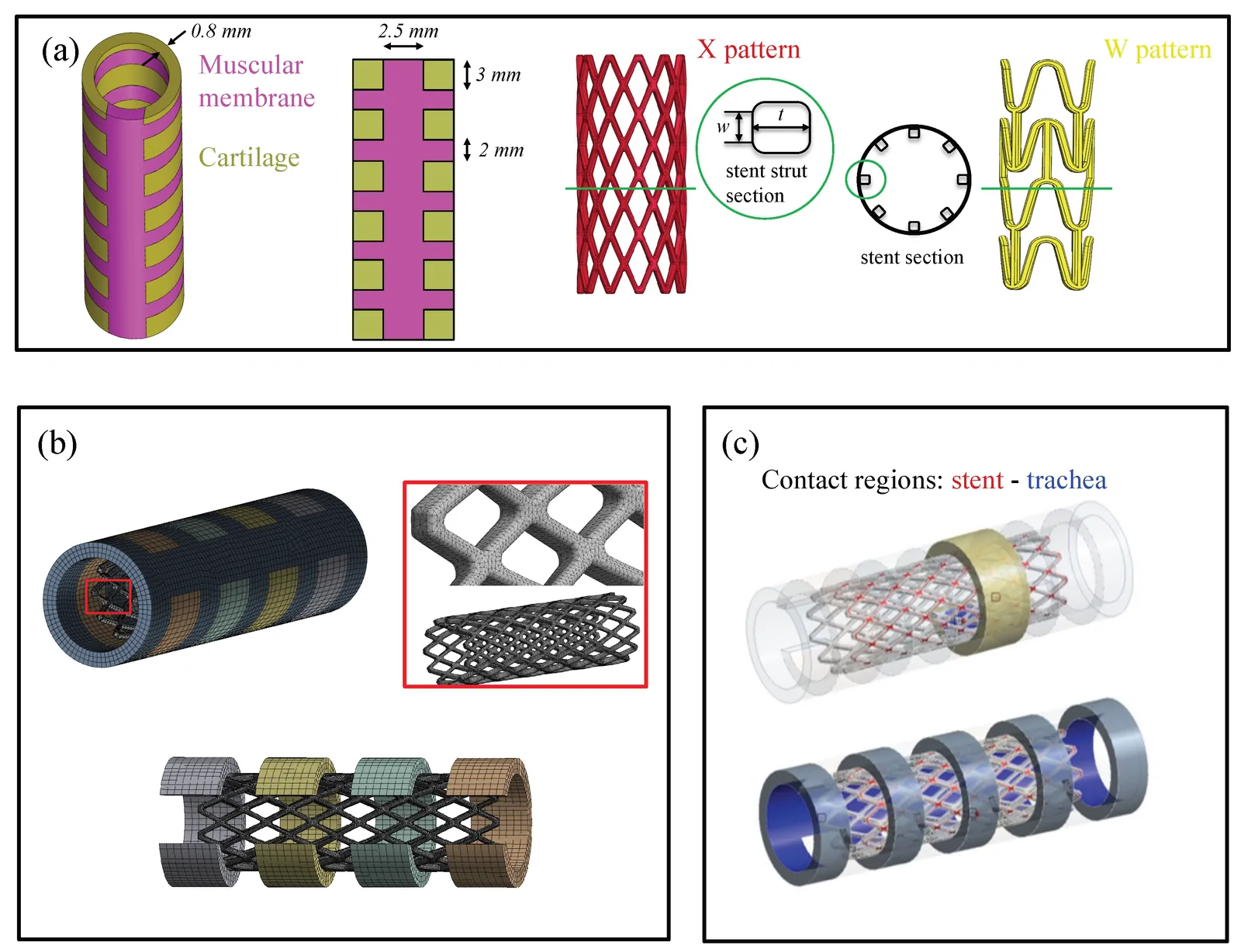
Computational models used in the study. (**a**) Rabbit trachea geometry, stent architectures (X-pattern and W-pattern), and definition of the principal geometric parameters, including wire thickness *t* and width *w*. (**b**) Finite element discretisation of the trachea and X-pattern stent. (**c**) Boundary conditions adopted during the deployment simulations.

The tracheal model was parameterised to allow modification of the principal airway dimensions, whereas the stent models were defined using two different lattice architectures, denoted as X-pattern and W-pattern, each requiring an independent geometric parameterisation because of their distinct structural layouts. These two representative designs were selected because they exhibit markedly different radial mechanical behaviour while both remaining compatible with extrusion-based additive manufacturing. For both stent patterns, the wire thickness *t* (Figure 1) was varied between 0.30 and 0.40 mm. Previous studies have shown that strut thickness is one of the primary parameters governing the radial stiffness of vascular stents, whereas strut width mainly affects the local contact pressure transmitted to the surrounding tissue [26]. Since the biodegradable stents considered in this work are manufactured by extrusion-based 3D printing, the strut cross-section was assumed to be square, and therefore the width and thickness were kept equal and varied simultaneously. Variations in cell shape and cell density were not considered in the present study in order to isolate the effects of stent architecture, material properties and wire thickness on the overall mechanical response.

### 2.1. Material Modelling

**Figure 2:**
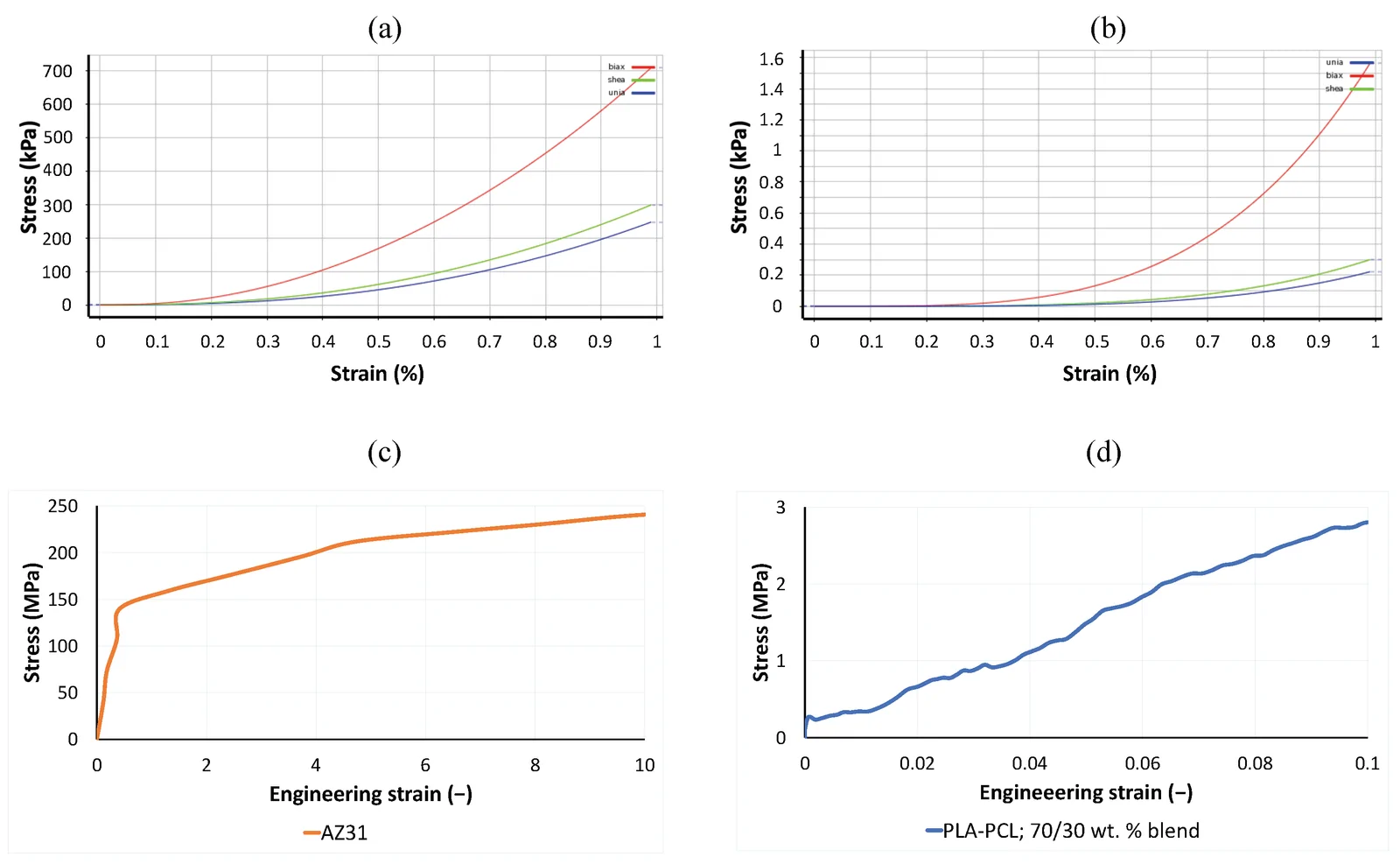
Constitutive material models employed in the numerical simulations. (**a**) Stress–strain response of rabbit tracheal cartilage. (**b**) Stress–strain response of the rabbit posterior muscular membrane. (**c**) Constitutive behaviour of the biodegradable AZ31 magnesium alloy. (**d**) Experimental constitutive behaviour of the PLA/PCL; 70/30 wt.% blend used for stent manufacturing.

The rabbit trachea was modelled as a heterogeneous structure composed of cartilaginous rings and the posterior muscular membrane. Both tissues were represented using a third-order Yeoh hyperelastic constitutive model, whose material parameters are summarised in Table 1. The corresponding stress–strain responses are shown in Figure 2a,b. The adopted constitutive models were identified from experimental mechanical tests previously performed on rabbit tracheal tissues and subsequently validated in earlier studies [24,25]. The geometry and main dimensions of the computational tracheal model are illustrated in Figure 1a.

Two biodegradable stent architectures were investigated, namely the X-pattern and the W-pattern (Figure 1b). For both designs, wire thicknesses ranging from 0.30 to 0.40 mm were considered, covering the typical dimensions achievable by extrusion-based additive manufacturing for biodegradable airway stents.

Two biodegradable materials were analysed. The first material was the biodegradable magnesium alloy AZ31. Its constitutive behaviour was defined from the engineering stress–strain curve reported by Grogan et al. [27], adopting an elastic modulus of E=44 GPa and a Poisson’s ratio of ν=0.35. Owing to its considerably higher elastic modulus, AZ31 provides substantially greater radial mechanical support than polymeric materials. Its corresponding engineering stress–strain curve is presented in Figure 2c.

The second material considered was a PLA/PCL; 70/30 wt.% blend because it provides an appropriate compromise between printability, mechanical performance and degradation behaviour for airway stent applications. Its constitutive behaviour was experimentally characterised by three-point bending tests performed on specimens manufactured using the same extrusion-based additive manufacturing process employed for the stents [28]. The measured mechanical properties are summarised in Table 2, while the corresponding engineering stress–strain curve is presented in Figure 2d. Since the initial portion of the experimental flexural response exhibited essentially linear elastic behaviour, the average flexural modulus obtained from three independent tests ($E_{f}=3.55$ GPa) was adopted as the elastic modulus of the constitutive model (E=3.5 GPa, ν=0.35).

Plastic deformation of both stent materials was modelled using the Multilinear Kinematic Hardening formulation available in ANSYS. The complete engineering stress–strain curves shown in Figure 2c,d were directly implemented as multilinear engineering stress–strain data. Consequently, the yield behaviour and subsequent hardening response were entirely defined by the corresponding constitutive curves, without prescribing additional analytical hardening parameters. Figure 2c,d show the constitutive curves over the strain range relevant to the mechanical response observed in the present simulations.

For completeness, the principal material properties are summarised in Table 3. Nevertheless, as aforementioned, the constitutive behaviour assigned to both stent materials in the finite element model was defined directly from the complete engineering stress–strain curves presented in Figure 2, using the multilinear kinematic hardening formulation available in ANSYS.

### 2.2. Stent Radial Crimping and Deployment

The deployment of the stent was simulated within a rabbit tracheal model with an internal diameter of 5 mm and a wall thickness of 0.8 mm (Figure 1a). The stent material behaviour was described using kinematic hardening plasticity, while frictionless contact was assumed between the stent and the tracheal wall (Figure 1c).

Stent expansion was displacement-controlled using a rigid cylindrical balloon positioned inside the device. Radial displacement was progressively applied to the balloon until the expanded stent reached a final diameter of 6 mm, producing contact with the tracheal wall. Although balloon-expandable stents are clinically deployed by controlling the inflation pressure, displacement control was adopted in the present work to prescribe the final stent diameter independently of the balloon mechanical response, following the approach proposed by Takashima et al. [29]. Prior to expansion, the balloon diameter was defined as being 0.1 mm smaller than the internal diameter of the undeployed stent. The balloon was modelled as a rigid cylinder with a Young’s modulus of 210 GPa and a Poisson’s ratio of 0.30, thereby preventing any significant balloon deformation during deployment.

Before analysing the interaction with the tracheal tissue, the radial mechanical behaviour of all stent configurations was characterised through numerical radial compression tests. A rigid cylindrical crimping head, whose internal diameter matched the external diameter of the undeployed stent, was displaced radially to compress the device from its initial diameter of 5 mm down to 3 mm, corresponding to a radial compression of 40%. The cylinder was subsequently unloaded until the compressive displacement was completely removed, allowing the complete loading–unloading response of the stent to be obtained. The initial stent diameter was selected according to the rabbit tracheal dimensions reported in previous studies [24]. Frictionless contact was assumed between the crimping head and the outer surface of the stent, while a remote displacement constraint was applied to a central node of the device to eliminate rigid body motion.

The reaction force acting on the crimping cylinder was recorded throughout the simulation to obtain the complete force–diameter hysteresis curves. These data were subsequently used to quantify the diameter recovery after radial compression, the compression force required to reach a representative diameter of 3.5 mm, and the local radial stiffness determined from the slope of the force–diameter curve within the corresponding compression interval. These complementary metrics were employed to compare the influence of stent architecture, material selection and wire thickness on the radial mechanical behaviour of the different stent configurations.

Following deployment inside the tracheal model, the distributions of maximum principal stress, maximum shear stress and peak values of maximum principal strain within the tracheal wall were extracted in order to characterise the mechanical interaction between the expanded stent and the surrounding tissue. In addition, the mean contact pressure at the stent–trachea interface was extracted directly from the finite element contact solution after deployment and used as an additional quantitative descriptor of the mechanical interaction between the stent and the airway wall.

All simulations were performed using ANSYS (Release 2020 R2, Ansys Inc., Canonsburg, PA, USA) on an HP Z2 G4 Workstation equipped with an Intel Core i9 processor (8 cores, 3.6 GHz) and 16 GB of RAM.

### 2.3. Meshing

The rabbit trachea was discretised with ANSYS IcemCFD (Release 2020 R2, Ansys Inc., Canonsburg, PA, USA) using structured eight-node hexahedral elements (Figure 1b). Three elements were distributed across the wall thickness in order to accurately capture the stress and strain gradients generated during stent deployment and tissue interaction. Owing to their complex lattice geometries, both the X-pattern and W-pattern stents were meshed using unstructured tetrahedral elements.

A mesh convergence study was performed to determine the appropriate element size for the stent models. Four different characteristic element sizes were evaluated (0.10, 0.09, 0.085, and 0.08 mm). The numerical results obtained with the three finest meshes differed by less than 1% for the main mechanical variables analysed, indicating mesh-independent solutions. Consequently, an element size of 0.09 mm was selected for all stent simulations, providing an appropriate compromise between numerical accuracy and computational efficiency.

The rigid expansion balloon employed during the deployment simulations was discretised using eight-node hexahedral elements. The resulting finite element meshes provided sufficient spatial resolution to accurately reproduce both the radial compression behaviour of the stents and their mechanical interaction with the surrounding tracheal wall.

### 2.4. Experimental Model

All experimental procedures were approved by the Animal Experimentation Ethics Committee of the Catholic University of Valencia (CEEAUCV2404), the CEEA, and the Instituto de Investigación Sanitaria (IIS) La Fe (2025-VSC-PEA-0066), Valencia, Spain.

The experimental investigation had two complementary objectives within the present study. First, the anatomical dimensions of the rabbit airway were used to define the computational tracheal model and the initial dimensions of the biodegradable stents. Second, the in vivo observations provided experimental evidence supporting the interpretation of the computational predictions regarding the mechanical interaction between the implanted stent and the surrounding tracheal tissue.

**Figure 3:**
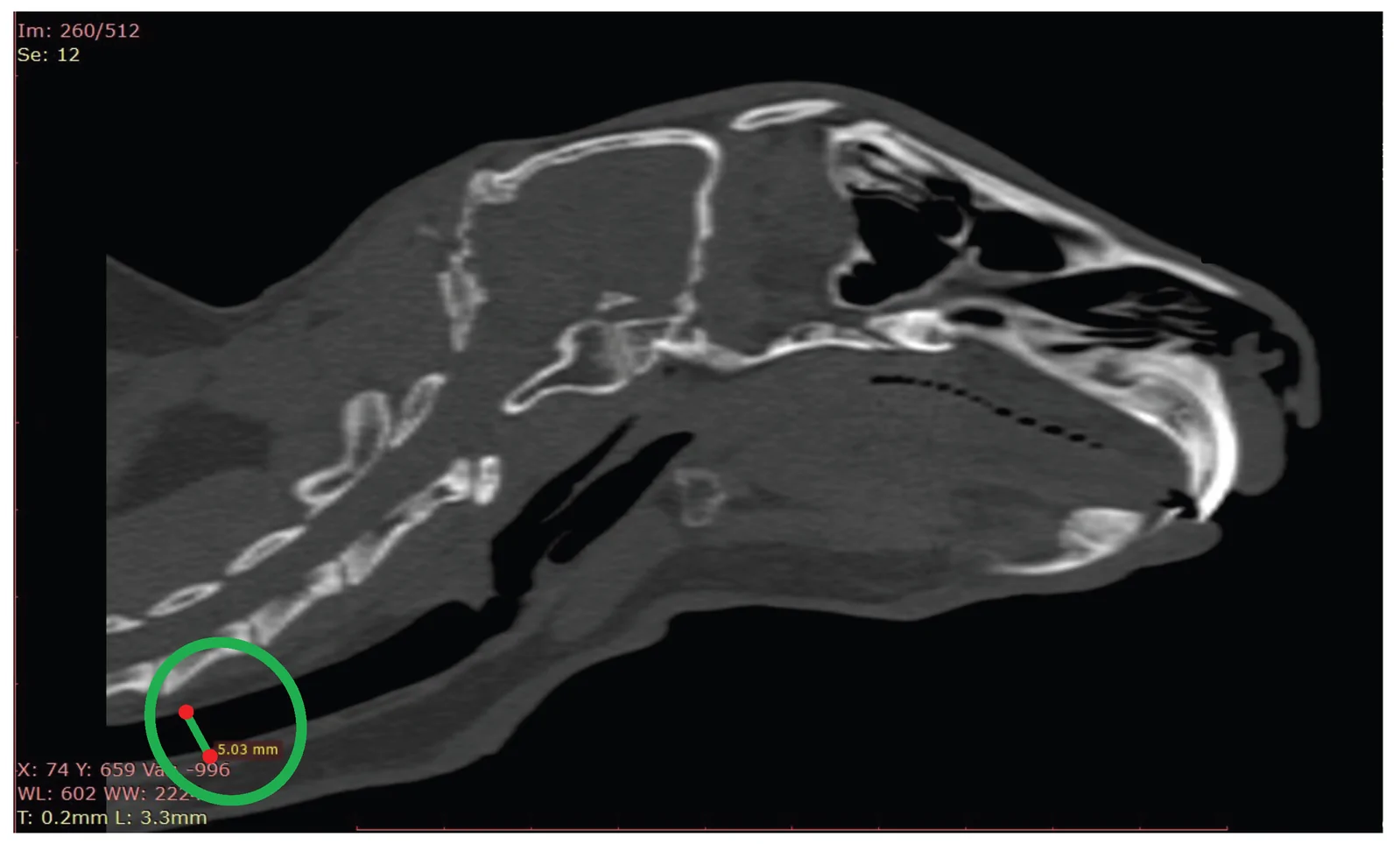
Representative computed tomography (CT) image of the rabbit trachea showing one of the measurements of the inner airway diameter. The measured lumen diameter (5.03 mm, green line inside the green circle) was used to parameterise the computational tracheal geometry and to define the initial diameter of the stent models employed in the numerical simulations.

The sample size was determined by means of an a priori statistical power analysis. Based on the tracheal damage score (TDS) reported by Novotny et al. [10], a 25% reduction in TDS (mean =3.53, standard deviation ± 0.5) was considered the minimum biologically relevant effect. Assuming a statistical power of 0.80 and a significance level of α=0.05, the analysis indicated that a minimum of four rabbits per follow-up group was required. In addition, one sham animal was included at each follow-up time point (4, 8 and 12 weeks) to account for the effect of the implantation procedure itself. Consequently, each follow-up group comprised five animals, resulting in a total of twelve implanted animals and three sham animals. To quantitatively assess the biological response following implantation, endoscopic findings were evaluated using the standardized scoring system proposed by Serrano-Casorrán et al. [30]. The assessment included four parameters, congestion, inflammation, secretions and stenosis, each graded as 0 (absence), 1 (moderate or present) or 2 (severe or abundant). The initial stent dimensions were established from anatomical measurements. A total of twelve rabbits were implanted with the selected biodegradable stent and underwent endoscopic follow-up examinations at 4, 8 and 12 weeks after implantation. As an example, a computed tomography (CT) scan acquired from one of the rabbits is presented in Figure 3. Airway dimensions were measured using the open-source software Horos™ (GNU Lesser General Public License, Version 3, LGPL-3.0, Annapolis, MD, USA). Measurements were performed along the cervical trachea, between the levels of the third and sixth cervical vertebrae (C3–C6), corresponding to the anatomical region selected for stent implantation. In addition, measurements obtained from the fifteen rabbits yielded a mean tracheal lumen diameter of 4.71±0.37 mm (mean ± standard deviation). Finally, a representative tracheal diameter of 5 mm was adopted in the computational model and subsequently used to define the initial dimensions of the stents analysed throughout this work.

Based on the computational analyses, X-pattern stents with a wire thickness of 0.35 mm were selected for the experimental study. This intermediate wire thickness was considered an appropriate compromise between radial mechanical support and structural flexibility. Compared with the 0.30 mm configuration, it provided greater radial resistance, whereas the 0.40 mm design exhibited greater stiffness without a substantial improvement in the overall mechanical response. Furthermore, the differences between adjacent wire thicknesses were relatively modest and remained within the practical dimensional tolerances associated with the additive manufacturing process. Consequently, the 0.35 mm configuration was considered a representative design for the preliminary in vivo evaluation.

The stents were manufactured by extrusion-based additive manufacturing using the same PLA/PCL; 70/30 wt.% blend, whose experimentally characterised mechanical properties were implemented in the numerical model, thereby ensuring consistency between the computational and experimental investigations.

The objective of the animal study was not to quantify the degradation kinetics of the material, which is being investigated in a separate ongoing study, but rather to evaluate the biological response associated with the mechanical interaction between the implanted stent and the tracheal tissue. During the first weeks following implantation, the devices largely preserved their structural integrity, allowing the observed tissue response to be interpreted predominantly in terms of the initial mechanical environment generated by the stent.

Consequently, the experimental observations were employed as a qualitative complement to the computational analyses rather than as a means of providing direct quantitative validation of the finite element model. Particular attention was paid to the spatial distribution of tissue alterations observed during endoscopic follow-up and their possible correspondence with the stress and strain patterns predicted numerically. Although no direct causal relationship can be established from the available data, the experimental observations provide encouraging evidence that the computational framework represents the main mechanical features governing biodegradable stent–tissue interaction and offers a plausible explanation for the biological response observed following implantation.

## 3. Results

### 3.1. Radial Compression Behaviour of the X and W Patterns for AZ31 and PLA/PCL; 70/30 Wt.% Blend

**Figure 4:**
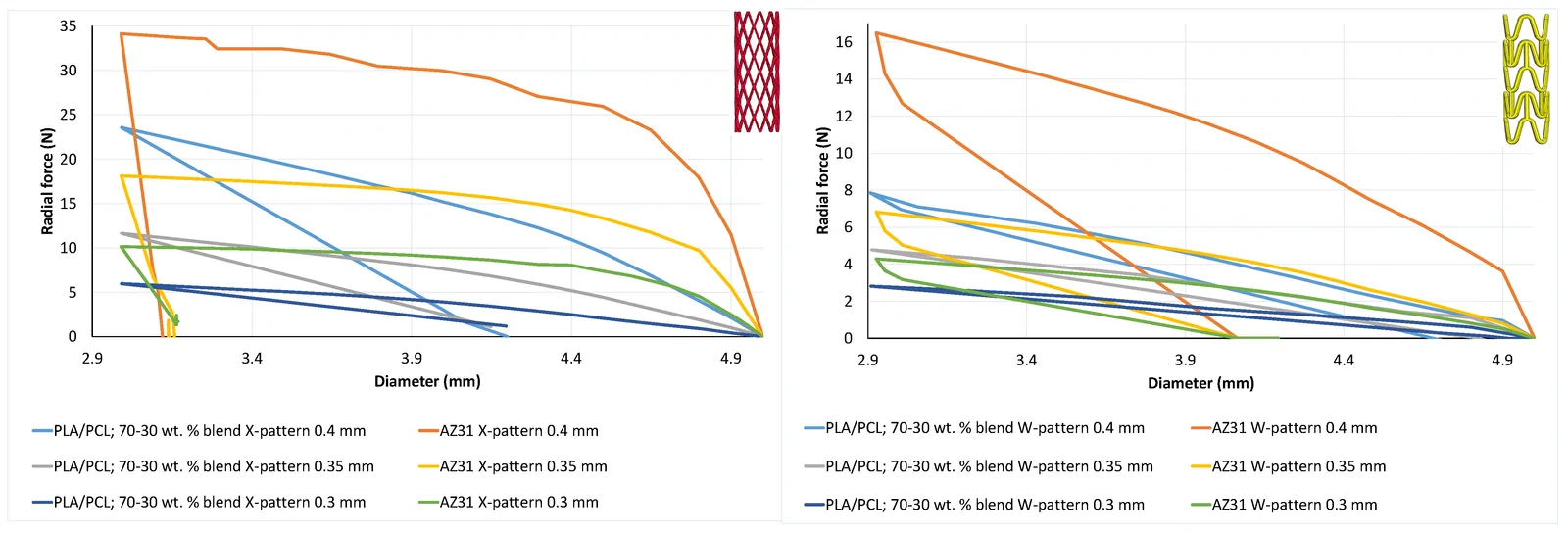
Comparison between the force–diameter curves of the stent designs with X and W patterns for AZ31 and PLA/PCL; 70/30 wt.% blend for different stent widths (0.4 mm, 0.35 mm and 0.3 mm).

Figure 4 compares the radial compression responses obtained for the X-pattern (left) and W-pattern stents (right) manufactured from AZ31 magnesium alloy and the PLA/PCL; 70/30 wt.% blend, respectively, for different wire thicknesses. To improve the readability of the force–diameter hysteresis curves, different vertical scales were adopted for the two subplots. The X-pattern curves are displayed using a vertical axis ranging from 0 to 35N, whereas the W-pattern curves are displayed using a vertical axis ranging from 0 to 16N. For all configurations analysed, the X-pattern required higher compressive forces than the corresponding W-pattern over the entire diameter range considered. This behaviour was consistently observed for both materials, indicating that stent architecture is the primary factor governing the radial mechanical response. The larger forces developed by the X-pattern demonstrate its greater resistance to radial deformation under external loading.

A clear influence of the material properties was also observed. For a given stent geometry and wire thickness, the AZ31 stents generally developed higher compressive forces than their PLA/PCL; 70/30 wt.% blend counterparts, particularly for the larger wire thicknesses. This behaviour is consistent with the considerably higher elastic modulus of AZ31 and reflects its greater load-bearing capacity during radial compression. In addition, the compressive force increased progressively with increasing wire thickness for both geometries and materials, as thicker struts provide greater structural resistance to radial deformation. This effect became particularly evident for the 0.40 mm configurations, where relatively small increases in wire thickness produced substantial increases in the force required to achieve the same diameter reduction.

The hysteresis curves obtained from the radial compression simulations also illustrate the influence of geometry and material on the elastic recovery of the stents after unloading. The AZ31 X-pattern exhibited the smallest diameter recovery after radial compression, recovering only a few tenths of a millimetre after compression from 5 mm to 3 mm. In contrast, the corresponding PLA/PCL; 70/30 wt.% blend X-pattern recovered to approximately 4.25 mm. Greater recovery was observed for the W-pattern for both materials. The AZ31 W-pattern recovered to approximately 4.0 mm, whereas the PLA/PCL; 70/30 wt.% blend W-pattern exhibited the largest elastic diameter recovery, recovering to nearly 4.8 mm after unloading. These results indicate that the X-pattern provides a more stable expanded configuration than the W-pattern, while the lower stiffness of the polymeric material leads to substantially greater elastic recovery than the metallic alternative.

Although the complete force–diameter hysteresis curves provide a comprehensive description of the radial compression behaviour, their nonlinear character makes direct quantitative comparisons between configurations less straightforward. For this reason, complementary quantitative metrics based on diameter recovery, radial stiffness and the compression force required to reach a representative diameter are presented in the following sections.

### 3.2. Quantitative Comparison of Elastic Diameter Recovery After Radial Compression

To further quantify the unloading behaviour observed in the hysteresis curves, the elastic diameter recovery of each stent configuration was calculated from the recovered diameter after removal of the compressive load. Elastic diameter recovery after radial compression is an important mechanical parameter for airway stents, as it reflects the ability of the device to recover its original geometry after transient radial compression and, consequently, to resist permanent deformation during external airway loading.

**Figure 5:**
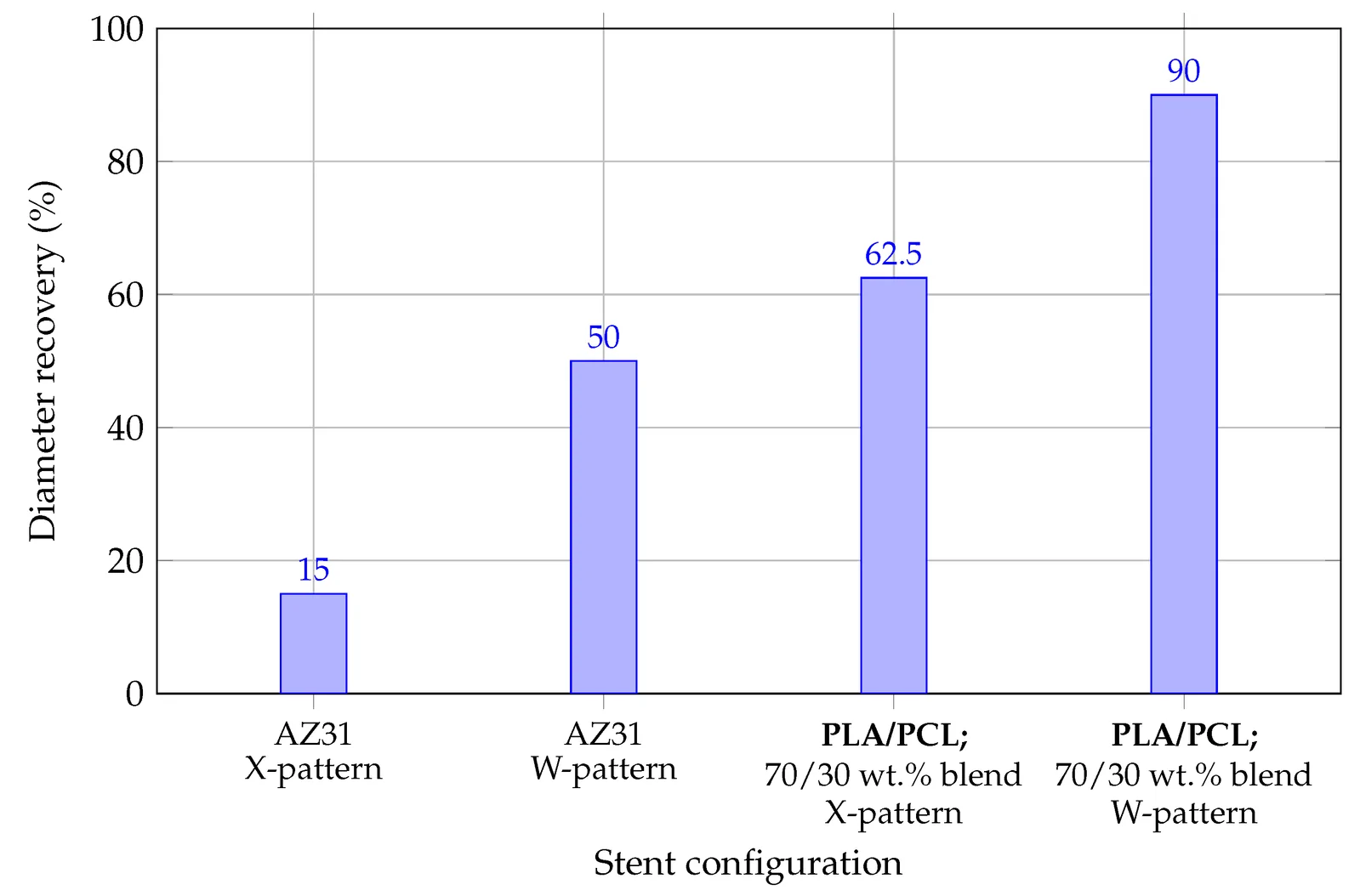
Diameter recovery after radial compression calculated from the recovered diameter after unloading during the radial compression simulations. Diameter recovery was defined as the percentage of the imposed diameter reduction recovered after removal of the compressive load. Higher values indicate greater elastic recovery, whereas lower values correspond to larger permanent deformation after compression. PLA/PCL denotes the poly(lactic acid)/poly(caprolactone) (70/30 wt.%) blend.

During the radial compression simulations, all stents were compressed from an initial diameter of 5 mm to 3 mm and subsequently unloaded. The diameter recovery was defined as

$$\mathrm{Diameter}\;\mathrm{recovery}\;\left(\%\right)=\frac{D_{\mathrm{recovered}}-D_{\mathrm{compressed}}}{D_{\mathrm{initial}}-D_{\mathrm{compressed}}}\times 100$$

where $D_{\mathrm{initial}}$ is the initial diameter prior to compression, $D_{\mathrm{compressed}}$ is the minimum diameter reached during loading, and $D_{\mathrm{recovered}}$ is the final diameter after unloading. The resulting diameter recovery values are summarised in Figure 5.

A clear influence of both stent geometry and material properties was observed. The AZ31 X-pattern exhibited the lowest diameter recovery after radial compression, recovering only approximately 15% of the imposed deformation after unloading. In contrast, the PLA/PCL; 70/30 wt.% blend W-pattern exhibited the highest diameter recovery after radial compression, recovering approximately 90% of the diameter reduction. Intermediate behaviour was observed for the AZ31 W-pattern and the PLA/PCL; 70/30 wt.% blend X-pattern, which exhibited diameter recovery values of approximately 50% and 62.5%, respectively.

These results demonstrate that both stent architecture and material properties strongly influence the elastic recovery of the device following radial compression. The W-pattern consistently exhibited greater diameter recovery than the corresponding X-pattern, while the polymeric PLA/PCL; 70/30 wt.% blend showed substantially greater recovery than the AZ31 stents owing to its lower stiffness and greater elastic compliance. Overall, these results provide a quantitative characterisation of the intrinsic structural response of the different stent configurations and complement the subsequent stent–tissue interaction analyses.

### 3.3. Quantitative Assessment of Radial Mechanical Performance

Although the complete force–diameter hysteresis curves provide a comprehensive description of the radial compression behaviour of the stents, their nonlinear character makes direct quantitative comparisons between configurations more difficult. Together with the diameter recovery after radial compression analysis presented above, two additional quantitative metrics were extracted from the compression simulations to characterise the radial mechanical performance of each design.

**Figure 6:**
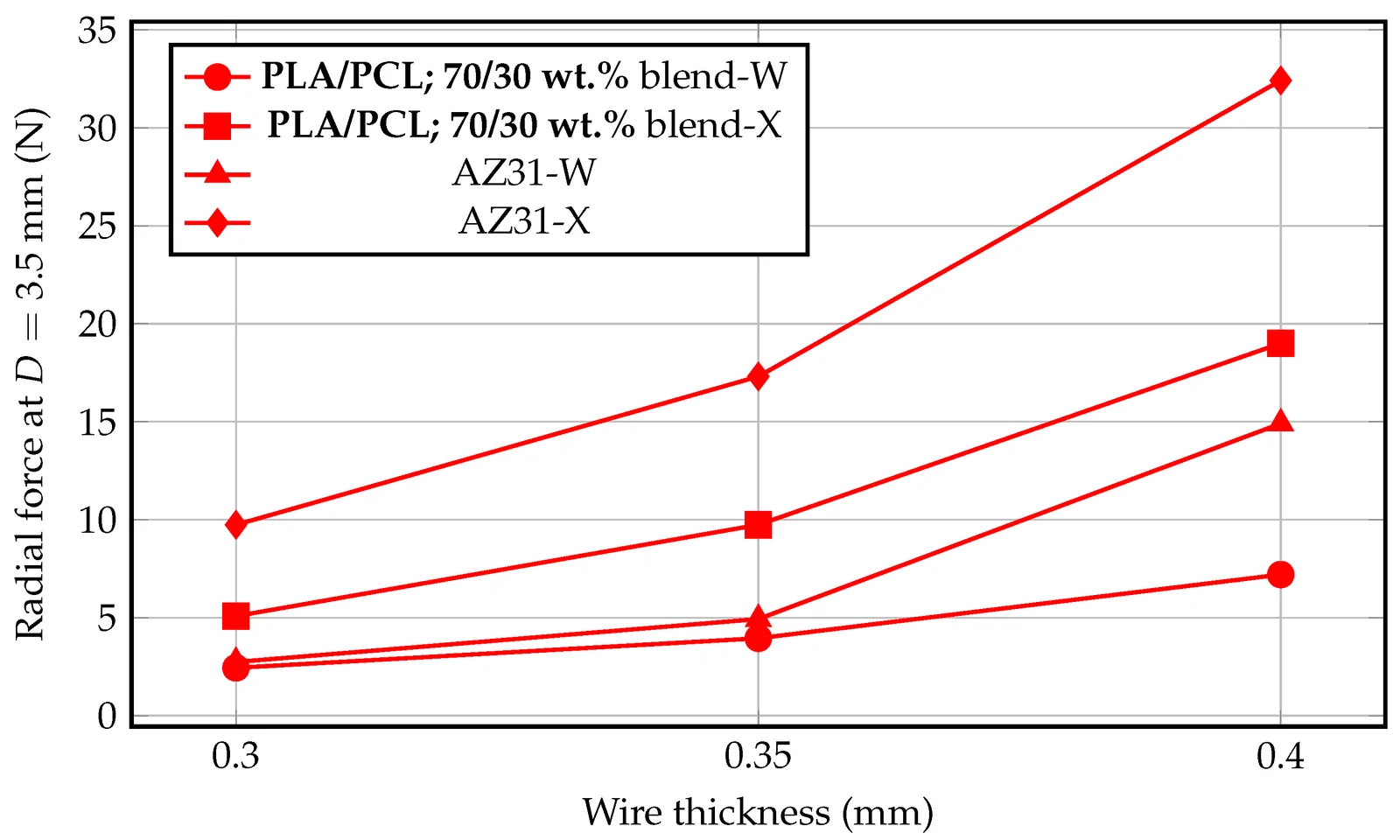
Radial force required to compress the different stent configurations to a diameter of 3.5 mm. The force increases with wire thickness for all configurations. The X-pattern consistently requires higher compressive forces than the W-pattern, while AZ31 exhibits a greater load-bearing capacity than PLA/PCL; 70/30 wt.% blend.

**Figure 7:**
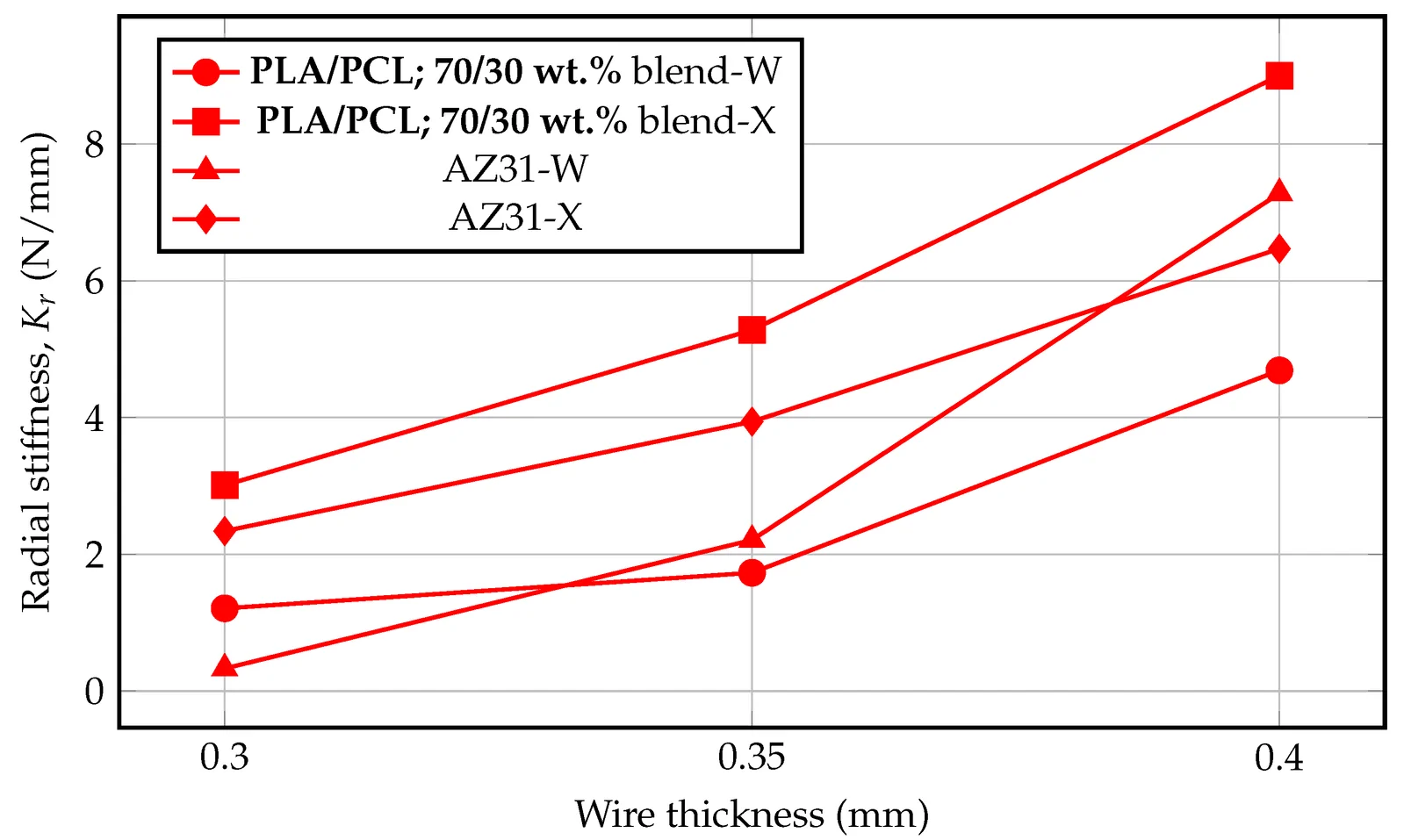
Radial stiffness calculated from the slope of the force–diameter response between D=4.5 mm and D=3.5 mm for the different stent configurations. The results show the influence of wire thickness, stent architecture and material properties on the resistance to radial deformation.

First, the radial force required to compress each stent to a representative diameter of 3.5 mm was evaluated (Figure 6). This parameter provides a direct measure of the radial load-bearing capacity of the device and facilitates comparison between different geometries, materials and wire thicknesses. Second, the local radial stiffness was calculated from the slope of the force–diameter response within the same diameter interval (Figure 7), providing complementary information on the local mechanical behaviour during compression.

The force required to reach a diameter of 3.5 mm revealed clear and consistent trends for all configurations (Figure 6). For both materials, the X-pattern required substantially higher compressive forces than the corresponding W-pattern, confirming the dominant influence of stent architecture on radial mechanical performance. Likewise, the AZ31 stents consistently developed higher compression forces than the PLA/PCL; 70/30 wt.% blend stents for a given geometry and wire thickness, reflecting the greater load-bearing capacity associated with the higher elastic modulus of the magnesium alloy. In addition, the compression force increased markedly with wire thickness for both stent architectures, indicating a progressive enhancement of radial support as the strut dimensions increased. Among all the configurations investigated, the AZ31 X-pattern with a wire thickness of 0.40 mm exhibited the greatest resistance to radial compression.

The radial stiffness analysis provided complementary information regarding the shape of the mechanical response (Figure 7). In general, radial stiffness increased with wire thickness for both stent architectures, and the X-pattern consistently exhibited higher stiffness values than the corresponding W-pattern. However, the influence of the material was less pronounced than that observed for the compression force. While AZ31 generally exhibited higher stiffness values for the larger wire thicknesses, the differences between AZ31 and the PLA/PCL; 70/30 wt.% blend progressively decreased as the wire thickness became smaller, and comparable local stiffness values were obtained for some configurations. This behaviour reflects the nonlinear character of the force–diameter response, in which the local slope does not necessarily follow the same trend as the overall compression force.

Taken together, the diameter recovery after radial compression, compression force and local radial stiffness provide a comprehensive quantitative description of the mechanical behaviour of the investigated stents. Diameter recovery characterises the ability of the device to elastically recover its original geometry following transient radial compression, the compression force at 3.5 mm quantifies its radial load-bearing capacity, and the local radial stiffness describes its resistance to deformation within a representative compression interval. Collectively, these parameters demonstrate that stent architecture is the primary factor governing radial mechanical performance, whereas material properties mainly influence the magnitude of the structural response. Wire thickness further modulates both effects, providing an additional design parameter for tailoring the mechanical behaviour of biodegradable airway stents.

### 3.4. Interaction Between Stents and Tissues

**Figure 8:**
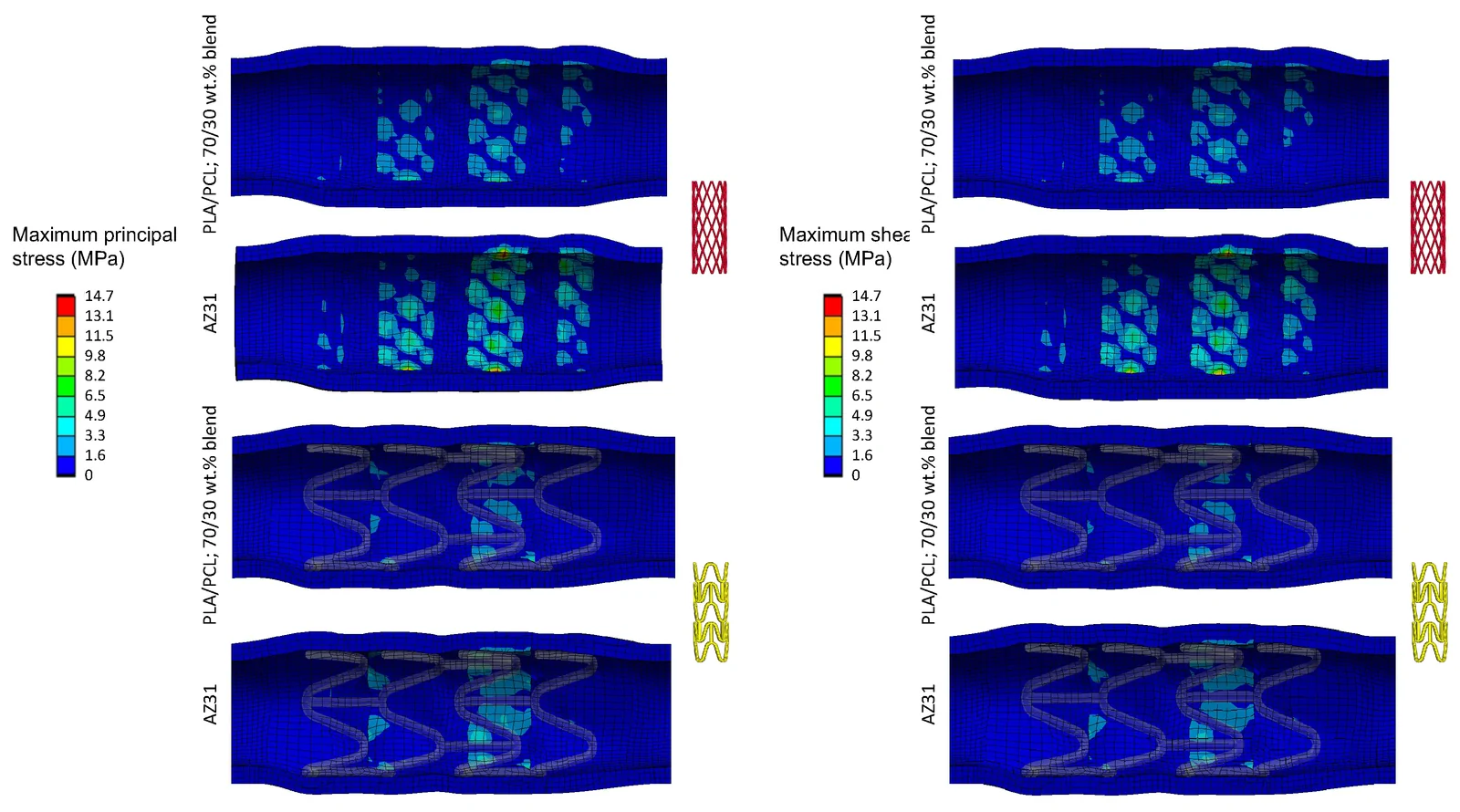
Comparison between the obtained maximum principal stress (**left panel**) and maximum shear stress (**right panel**) after stent deploying for the stent designs with X and W patterns with wire thickness 0.35 mm and materials AZ31 and PLA/PCL; 70/30 wt.% blend.

The mechanical interaction between the expanded stents and the rabbit tracheal tissue was evaluated through the distributions of maximum principal stress, maximum shear stress and maximum principal strain predicted by the finite element simulations. Figure 8 compares the mechanical fields generated after deployment for both stent architectures and materials. Since the different wire thicknesses produced essentially identical spatial distributions, differing only in the magnitude of the predicted stresses, only the representative configuration with a wire thickness of 0.35 mm is presented.

Figure 8 illustrates the distributions of maximum principal stress and maximum shear stress within the tracheal wall. A clear influence of stent geometry was observed. For both materials, the X-pattern generated more extensive regions of elevated stress than the corresponding W-pattern. The characteristic arrangement of the X-pattern produced a more homogeneous distribution of the contact loads throughout the tracheal wall, whereas the W-pattern generated more localised stress concentrations around individual struts.

The material mainly affected the magnitude of the stresses rather than their spatial distribution. For a given stent geometry, AZ31 and the PLA/PCL; 70/30 wt.% blend produced remarkably similar stress patterns, indicating that geometry primarily determines where the mechanical loads are transmitted to the tissue. However, the considerably higher stiffness of AZ31 resulted in substantially larger stress levels owing to its lower diameter recovery and greater radial support. Maximum principal stresses approached 15 MPa for the AZ31 configurations, whereas peak values of approximately 5 MPa were obtained for the corresponding PLA/PCL; 70/30 wt.% blend stents. Similar trends were observed for the maximum shear stress distributions. These results indicate that geometry defines the spatial footprint of the mechanical interaction, while material properties govern the intensity of the stresses transmitted to the airway tissue.

The distributions of maximum principal strain followed trends similar to those observed for the stress fields. Since they did not provide any additional information regarding the mechanical interaction between the stent and the tracheal wall, the corresponding contour maps are not presented. Nevertheless, the numerical results showed that the X-pattern generated a more extensive deformation field, with local maximum principal strain values reaching approximately 90% in regions immediately adjacent to the stent struts, whereas the W-pattern produced lower maximum principal strain values of approximately 65%. These results further support the conclusion that the stent architecture primarily governs the deformation pattern of the tracheal wall following deployment. Overall, the simulations demonstrate that stent geometry and material properties play complementary mechanical roles during deployment. The stent architecture primarily governs the spatial distribution of stresses and strains within the tracheal wall, whereas material properties determine the magnitude of the mechanical response and the final airway opening achieved after expansion. Consequently, both factors should be considered simultaneously during the design of biodegradable airway stents intended to provide adequate radial support while limiting excessive tissue loading.

To complement the qualitative interpretation of the stress contour maps, the mean contact pressure between the stent and the tracheal wall was also evaluated for the 0.35 mm wire-thickness configurations (Table 4). The AZ31 stents generated substantially higher mean contact pressures than the corresponding PLA/PCL; 70/30 wt.% blend configurations owing to their greater stiffness. In addition, the X-pattern consistently produced higher mean contact pressures than the W-pattern for both materials, confirming that both material properties and stent architecture contribute to the mechanical interaction with the airway wall.

### 3.5. In Vivo Observations and Preliminary Qualitative Comparison with Computational Findings

Based on the radial compression, elastic diameter recovery and tissue interaction analyses, the X-pattern stent was selected for the in vivo evaluation. Compared with the W-pattern configuration, the X-pattern exhibited greater resistance to radial compression, lower elastic diameter recovery and a superior ability to maintain its expanded configuration after deployment during in silico testing, suggesting an improved capability to preserve airway patency. Consequently, this design was considered the most suitable candidate for experimental implantation. Although the AZ31 stents exhibited superior radial mechanical performance, the PLA/PCL; 70/30 wt.% blend stents were selected for the animal study owing to their greater manufacturing feasibility by extrusion-based additive manufacturing, lower production cost, and more suitable degradation profile for the planned in vivo evaluation. The implanted devices were manufactured from the same PLA/PCL; 70/30 wt.% blend whose mechanical properties were experimentally characterised and subsequently implemented in the numerical model. Therefore, consistency was maintained between the computational material model and the experimental devices. Based on the numerical results, X-pattern stents with a wire thickness of 0.35 mm were manufactured and implanted in twelve rabbits. Endoscopic follow-up examinations were performed at 4, 8 and 12 weeks after implantation.

**Figure 9:**
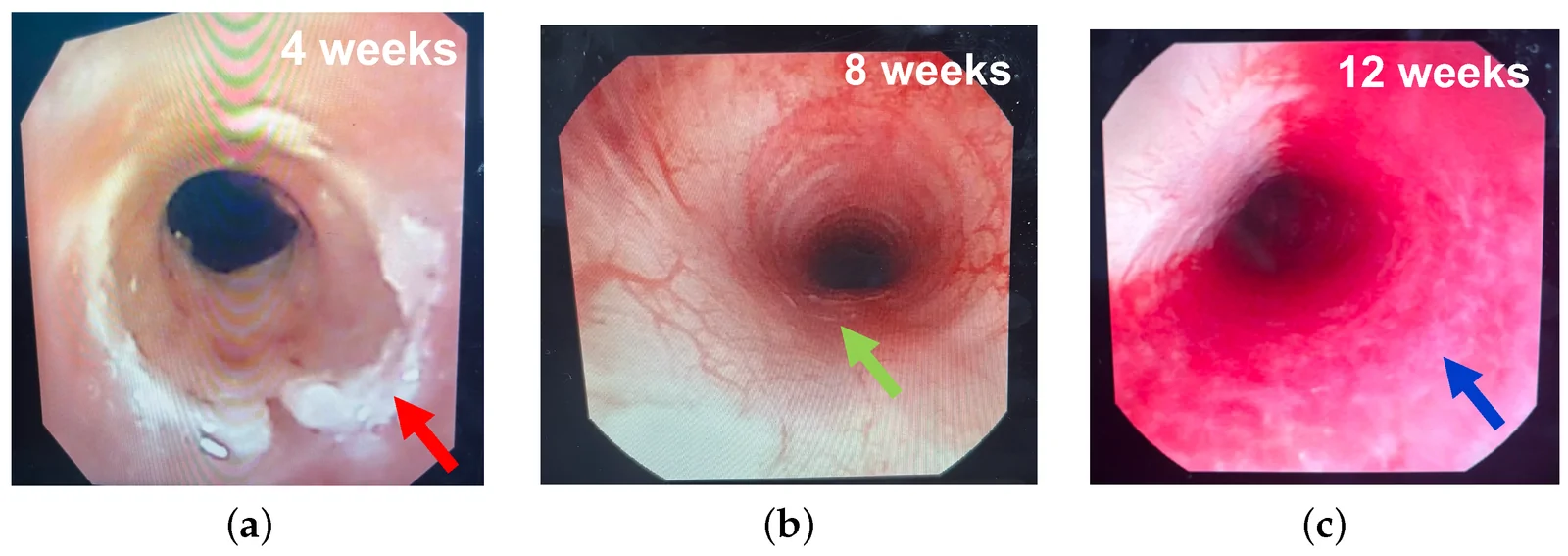
Representative endoscopic observations obtained during follow-up after the implantation of PLA/PCL; 70/30 wt.% blend X-pattern tracheal stents in rabbits. (**a**) Intraluminal stent at the 4-week follow-up; localized tissue remodeling and small mucus accumulations are visible near the proximal implantation region (red arrow), corresponding to regions where the computational model predicted relatively high strain levels. (**b**) Normal tracheal tissue after the 8-week follow-up, with the presence of mucus (green arrow). (**c**) Hyperemic tracheal wall and presence of mucus after the 12-week follow-up, exhibiting a spatial arrangement reminiscent of the original X-pattern architecture (blue arrow).

Representative endoscopic observations are presented in Figure 9. Figure 9a corresponds to the 4-week follow-up, Figure 9b to the 8-week follow-up, and Figure 9c corresponds to the 12-week follow-up. At 8 weeks, the implanted stents were no longer visible within the tracheal lumen, and at 12 weeks, only one rabbit still exhibited a visible stent, indicating complete degradation of the polymeric structure or expectoration before the follow-up. Nevertheless, characteristic surface features remained visible on the tracheal wall.

In particular, Figure 9a shows a trachea with a stent undergoing degradation, where small accumulations of mucus together with localised tissue changes are visible near the proximal implantation region (red arrow). These features appear to coincide with the regions where the computational model predicted the greatest local tissue strains. The numerical simulations indicated that the X-pattern generated maximum principal strain values of approximately 0.9 (90% strain) in regions immediately adjacent to the stent struts, whereas the W-pattern produced lower maximum values of approximately 0.65 (65% strain). These regions therefore represent the locations where the greatest tissue deformation is expected during stent deployment.

Figure 9b shows normal tracheal tissue with residual mucus (green arrow). Overall, the experimental observations should be interpreted as a qualitative comparison with the computational predictions rather than as a direct validation of the finite element model. Nevertheless, the endoscopic findings are broadly consistent with the spatial mechanical trends predicted numerically, supporting the hypothesis that stent geometry, radial mechanical performance and the resulting local mechanical environment collectively influence the subsequent response of the tracheal wall following implantation.

Figure 9c shows a hyperaemic reaction (indicated by the blue arrow) and mucus. Although the biological mechanisms responsible for this appearance cannot be determined from the present study, the observed morphology is compatible with local tissue remodelling or endoscopically observed tissue changes occurring in regions previously occupied by the stent. Interestingly, the spatial distribution of these tissue features appears qualitatively consistent with the mechanical environment predicted by the computational simulations. As shown in Figure 8, the X-shaped pattern generated characteristic distributions of maximum principal stress within the tracheal wall, with elevated stress regions extending between adjacent struts and along the supporting structure. Although no direct causal relationship can be established, the computational results suggest that the local mechanical environment generated by the stent may have influenced the tissue morphology observed during the endoscopic follow-up.

**Figure 10:**
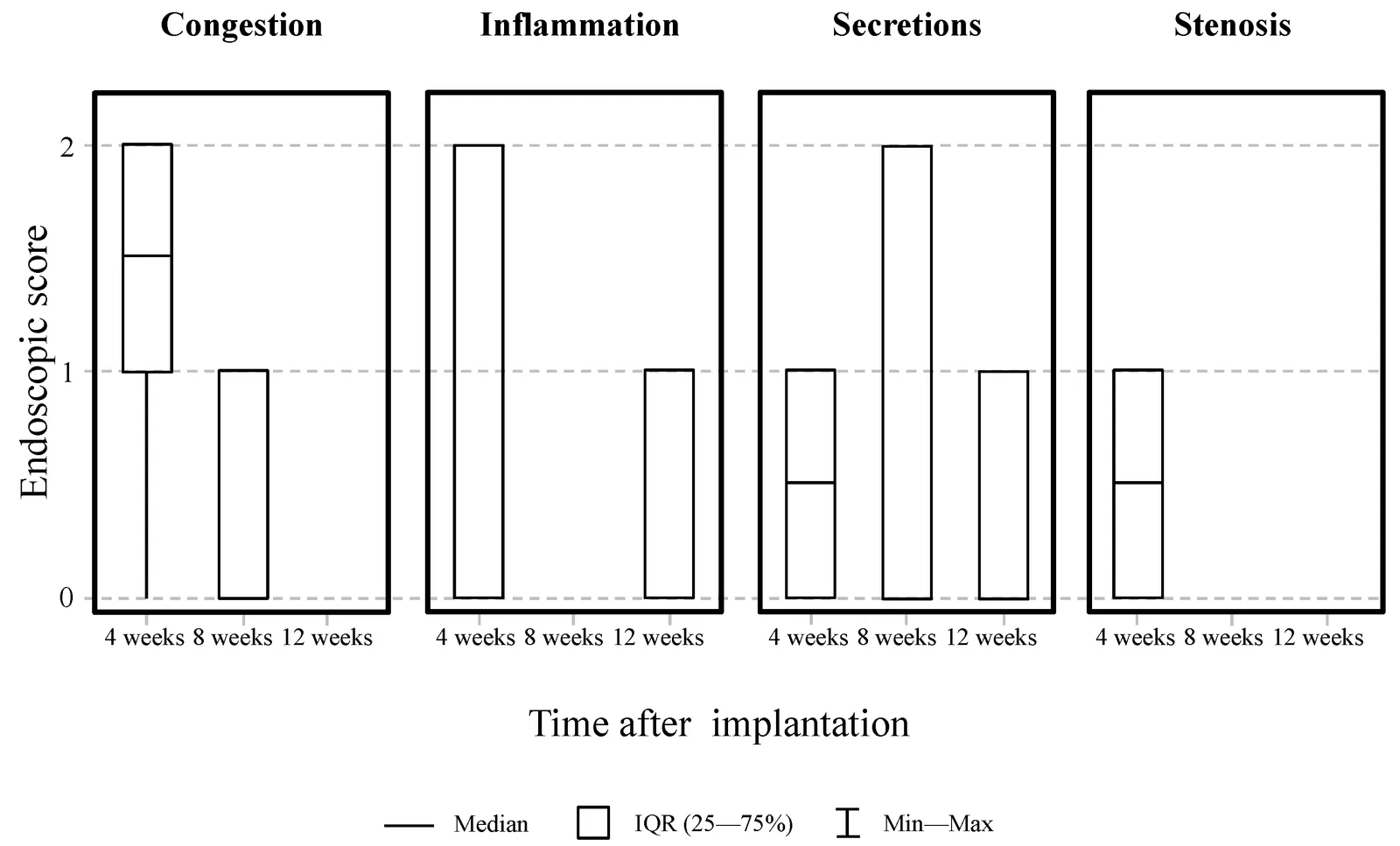
Semi-quantitative endoscopic assessment performed at 4, 8 and 12 weeks after implantation. Horizontal bars represent the median endoscopic score and vertical bars indicate the minimum–maximum range. The numbers below each marker indicate the corresponding follow-up time (weeks). Granuloma formation was not observed in any animal during the study and is therefore not included.

To complement the qualitative interpretation of the representative endoscopic images, a semi-quantitative endoscopic assessment was performed for all animals at each follow-up time point using the scoring system described in Section 2.4. The corresponding results are summarised in Figure 10, where the horizontal bars indicate the median score and the vertical bars represent the minimum–maximum range for each evaluated parameter. Congestion showed relatively high scores throughout the follow-up period, with a slight decrease at 8 weeks and higher values at 12 weeks. Inflammation followed a similar trend, with minimal scores at 8 weeks and increasing again at the final evaluation. Secretions remained generally mild throughout the follow-up period, although greater variability was observed at 8 weeks. Stenosis scores were consistently low and tended to decrease over time, indicating that no clinically relevant airway narrowing developed after implantation. No granuloma formation was detected in any animal throughout the study; therefore, this parameter is not included in Figure 10. Overall, the quantitative endoscopic evaluation supports the qualitative observations presented above and indicates that the implanted biodegradable stents produced only mild tissue alterations during the follow-up period.

## 4. Discussion

The use of tracheobronchial stents for the treatment of airway pathologies remains challenging because of the high incidence of complications and the frequent need for reintervention [13]. Silicone stents are associated with migration and mucus obstruction [31], whereas metallic devices may induce excessive tissue reactions, including inflammation, granulation tissue formation and abnormal cellular proliferation [32]. Despite continuous developments in silicone stent technology [17,22,33,34,35], important limitations remain. Furthermore, although metallic stents can be implanted using flexible bronchoscopy under conscious sedation [2], silicone stents generally require rigid bronchoscopy and general anaesthesia [31]. These limitations continue to motivate the development of biodegradable alternatives capable of providing temporary mechanical support while avoiding the long-term complications associated with permanent implants.

Although biodegradable airway stents have shown encouraging clinical and experimental results [7,10,11,12,36,37], their mechanical behaviour remains considerably less understood than in cardiovascular applications, where extensive computational and experimental studies have demonstrated the importance of optimising both stent geometry and material properties to achieve an appropriate balance between radial support and tissue loading [38,39,40]. Similar systematic investigations are still scarce in airway stenting despite the fact that mechanical support and tissue interaction are fundamental determinants of clinical performance. The present work contributes to filling this gap by providing a computational framework that simultaneously evaluates radial compression, diameter recovery and stent–tissue interaction, thereby offering a more comprehensive mechanical assessment than radial stiffness alone.

The present computational framework represents the mechanical conditions immediately after stent deployment, when the biodegradable device provides its maximum radial support. As degradation progresses, polymer chain scission and material resorption progressively reduce the stiffness and radial force of the stent, thereby modifying the stress transfer to the surrounding airway tissue and the ability of the device to maintain lumen patency. The degradation rate therefore becomes a key design parameter, since excessively rapid degradation may result in premature loss of mechanical support, whereas excessively slow degradation may prolong the presence of a foreign body and increase the risk of complications. Previous experimental and clinical studies have highlighted that achieving an appropriate balance between mechanical stability and degradation kinetics remains one of the principal challenges in the development of biodegradable airway stents [13,36,37]. Although these time-dependent phenomena were beyond the scope of the present study, they represent an important direction for future computational developments.

Rather than identifying a single optimal design, the numerical analyses demonstrated that the mechanical behaviour of biodegradable airway stents results from the combined effects of geometry, material properties and nonlinear structural deformation. In particular, the superior radial performance of the X-pattern can be attributed to its structural architecture. The inclined struts create a more continuous load-transfer path around the circumference of the stent, promoting a more uniform distribution of internal forces during radial compression. In contrast, the longer unsupported segments of the W-pattern undergo greater bending deformation, resulting in lower radial stiffness and greater diameter recovery after unloading. Material selection primarily affected the magnitude of the mechanical response, with the higher elastic modulus of AZ31 producing greater radial resistance and reduced diameter recovery compared with the PLA/PCL; 70/30 wt.% blend. These observations indicate that geometry governs the deformation mechanism, whereas material properties mainly determine the intensity of the structural response.

Few computational studies have simultaneously analysed radial compression, diameter recovery and tissue interaction in biodegradable airway stents. Direct quantitative comparisons are difficult because most published studies focus on experimental implantation or global radial compression tests rather than detailed computational analyses of stent–tissue interaction. However, the trends observed in the present work are consistent with previous reports. Experimental studies have consistently shown that increasing structural stiffness improves airway support at the expense of increasing the mechanical interaction with the surrounding tissue [7,36,37]. Similarly, computational investigations in cardiovascular stents have demonstrated that geometry governs the distribution of stresses whereas material stiffness mainly affects their magnitude [38,39,40], which is in agreement with the behaviour observed in the present airway models.

The tissue interaction analyses further demonstrated that geometry and material influence different aspects of the mechanical environment generated within the tracheal wall. Whereas the stent architecture primarily determined the spatial distribution of stresses and strains, the material stiffness primarily controlled their magnitude. These observations also illustrate that the design of biodegradable airway stents involves balancing sufficient radial support to maintain airway patency against limiting the mechanical loading transmitted to the surrounding tissue. Consequently, the selection of the experimental configuration was based on the combined assessment of radial compression behaviour, diameter recovery, airway expansion and tissue interaction rather than on maximising a single mechanical parameter. The X-pattern with a wire thickness of 0.35 mm was therefore selected as a representative compromise between radial support and structural flexibility. Compared with the 0.30 mm configuration, it provided greater radial resistance, whereas the 0.40 mm configuration exhibited higher stiffness without a substantial improvement in the overall mechanical response. Moreover, the differences between adjacent wire thicknesses were relatively modest and remained within the practical dimensional tolerances associated with the adopted additive manufacturing process.

The computational predictions were further complemented by in vivo observations obtained from rabbit implantation experiments using X-pattern stents manufactured from the same PLA/PCL; 70/30 wt.% blend characterised experimentally and implemented in the numerical model. Although the animal study was not intended to provide a quantitative validation of the finite element simulations, the endoscopic observations are consistent with the hypothesis that the local mechanical environment established immediately after implantation may influence the subsequent biological response of the tracheal wall. Surface imprints resembling the original stent architecture remained visible after degradation of the device, while localised tissue alterations qualitatively corresponded to regions of elevated stresses and strains predicted numerically. Although no causal relationship can be established from the available data, these observations reinforce the importance of incorporating tissue–stent interaction analyses into the design process of biodegradable airway prostheses.

From a translational perspective, the present results indicate that the selection of biodegradable airway stents should not rely exclusively on degradation behaviour or material properties. Instead, radial support, elastic diameter recovery, lumen maintenance and tissue loading should be considered simultaneously to achieve an appropriate mechanical balance between airway stabilisation and biological compatibility. Unlike cardiovascular biomechanics, where optimisation strategies and patient classifications have already been proposed to support device selection [41], airway stenting still lacks objective biomechanical design criteria, and stent selection frequently depends on clinical experience [13]. Consequently, rather than proposing a unique optimal configuration, the present work establishes a systematic computational methodology capable of evaluating the influence of geometry, material properties and design parameters on the mechanical behaviour of biodegradable airway stents. Such a framework represents an important step towards the future development of patient-specific biodegradable airway prostheses based on objective biomechanical criteria.

### Limitations of the Study

Several limitations of the present study should be acknowledged. First, although preliminary in vivo experiments were performed, the animal study involved a limited number of subjects and was intended primarily as a qualitative comparison with the computational predictions. Consequently, the agreement between the endoscopic observations and the simulated mechanical variables should not be interpreted as a direct quantitative validation of the numerical model.

Second, the computational framework was intentionally limited to the initial post-deployment stage, since the primary objective of this work was to investigate how stent geometry, material properties and design parameters influence the initial mechanical environment experienced by the tracheal wall. Time-dependent phenomena such as material degradation, changes in mechanical properties during degradation, tissue remodelling, inflammation and biological adaptation were not incorporated into the present simulations. Since these processes may significantly influence the long-term behaviour of biodegradable airway stents, their inclusion constitutes an important objective of future developments.

Furthermore, several modelling assumptions were adopted to obtain a computationally efficient framework suitable for design exploration. The tracheal geometry and material behaviour were simplified, and physiological phenomena such as respiratory motion, mucus accumulation and patient-specific variability were not explicitly considered. Likewise, the contact interaction was assumed to be frictionless, representing a simplified approximation of the mechanical interaction between the stent and the airway mucosa. Consequently, the numerical results should be interpreted as a comparative analysis of different stent designs rather than as an exact prediction of the clinical outcome.

A frictionless contact formulation was adopted at the stent–trachea interface. In clinical conditions, friction and local tissue adhesion may partially restrict axial sliding and modify the local distribution of contact stresses. Consequently, although the frictionless assumption may influence the local contact pressure distribution and the extent of relative motion between the stent and the airway wall, it is not expected to alter the overall comparative trends observed between the different stent geometries and materials, since all configurations were analysed under identical contact conditions. Moreover, the tracheal model was based on an idealised rabbit geometry rather than on patient-specific anatomy. Anatomical variability, local wall thickness and regional curvature may influence the absolute magnitudes of tissue stresses and strains. Nevertheless, the objective of the present work was to establish a systematic computational framework for comparative evaluation of stent design parameters under controlled conditions rather than to reproduce the mechanical response of a specific subject. Future developments should incorporate patient-specific geometries together with more realistic contact conditions to further improve the predictive capability of the model.

Finally, although the proposed methodology is fully compatible with patient-specific modelling, no formal optimisation procedure was performed. The objective of the present work was to systematically investigate the influence of stent geometry, material properties and design parameters on the mechanical response of biodegradable airway stents rather than to identify a unique optimal configuration. In contrast to cardiovascular biomechanics, where patient classifications and optimisation strategies have been proposed to support device design [41], airway stenting still lacks well-established optimisation criteria. In current clinical practice, the selection of stent dimensions and characteristics frequently relies on physician experience [13]. The computational framework presented here therefore constitutes a first step towards the future optimisation and personalisation of biodegradable airway prostheses based on objective biomechanical criteria.

Despite these limitations, the proposed methodology provides a systematic framework for evaluating the mechanical performance of biodegradable airway stents by integrating computational modelling, experimentally characterised biomaterials and preliminary in vivo observations. The approach contributes to a better understanding of the mechanical factors governing stent–tissue interaction and establishes a solid foundation for the future development and optimisation of patient-specific biodegradable airway devices.

## 5. Conclusions

A computational framework has been developed to investigate the mechanical interaction between biodegradable airway stents and the tracheal wall, providing a systematic methodology for analysing the influence of stent geometry, material properties and design parameters on device performance. By integrating computer-aided design, finite element modelling and experimentally characterised biodegradable materials, the proposed approach enables the evaluation of customised airway stent configurations prior to implantation.

The numerical results demonstrated that both stent geometry and material properties strongly influence the mechanical response of biodegradable airway stents, although they affect different aspects of the stent–tissue interaction. The X-pattern consistently exhibited greater resistance to radial compression, lower diameter recovery after radial compression and improved maintenance of airway patency compared with the W-pattern. Stent architecture primarily influenced the spatial distribution of stresses and strains within the tracheal wall, whereas material properties mainly determined the magnitude of the mechanical response. This interpretation was further supported by the quantitative contact-pressure analysis, which confirmed the influence of both stent geometry and material stiffness on the mechanical interaction with the airway wall. Among the investigated materials, AZ31 provides the greatest radial mechanical support, whereas the PLA/PCL; 70/30 wt.% blend represents a suitable biodegradable polymeric alternative compatible with extrusion-based additive manufacturing.

The computational analyses guided the selection of the X-pattern PLA/PCL; 70/30 wt.% blend stent for the experimental study. Preliminary in vivo observations in rabbits showed tissue features that were qualitatively consistent with the stress and strain distributions predicted numerically, although no direct causal relationship can be established from the available data. These findings suggest that the proposed computational framework adequately captures relevant aspects of the mechanical interaction between the implanted device and the surrounding airway tissue.

Overall, the proposed methodology provides a practical tool for the rational design and mechanical assessment of biodegradable airway stents and represents a first step towards the future optimisation and personalisation of airway prostheses. Future work will incorporate degradation phenomena, tissue remodelling, standardised quantitative biological assessment, and larger experimental datasets to further improve the predictive capability and clinical applicability of the computational framework.

## Figures and Tables

**Table 1:** Material parameters employed for the third-order Yeoh hyperelastic constitutive model describing the rabbit tracheal tissues. The parameters $C_{10}$, $C_{20}$ and $C_{30}$ correspond to the Yeoh material constants, whereas *d* defines the compressibility parameter.

Parameters	$C_{10}$ [MPa]	$C_{20}$ [MPa]	$C_{30}$ [MPa]	*d* [MPa^−1^]
Cartilage	0.51858	17.94582	0.024344	51.8585
Muscular membrane	0.0042342	0.0802505	0.525712	42.34

**Table 2:** Mechanical properties of the PLA/PCL; 70/30 wt.% blend obtained from three-point bending tests performed on three specimens manufactured using the same additive manufacturing process employed for the stents.

Specimen	$E_{f}$ (MPa)	$\sigma_{f,\max}$ (MPa)	$\varepsilon_{f,\max}$ (%)
PLA/PCL; 70/30 wt.% 1	4135.15	29.69	2.07
PLA/PCL; 70/30 wt.% 2	3335.35	28.00	2.42
PLA/PCL; 70/30 wt.% 3	3181.98	32.45	3.55
Mean ± SD	3550.83±514.72	30.05±2.25	2.68±0.77

**Table 3:** Mechanical properties and constitutive formulations assigned to the stent materials in the finite element model.

Material	*E* (GPa)	ν	$\sigma_{y}$ (MPa)	Constitutive Formulation
PLA/PCL; 70/30 wt.%	3.5	0.35	30.0	Multilinear kinematic hardening
AZ31 magnesium alloy	44.0	0.35	190	Multilinear kinematic hardening

**Table 4:** Mean contact pressure between the stent and the tracheal wall for the 0.35 mm wire-thickness configurations.

Material	Stent Architecture	Mean Contact Pressure (MPa)
AZ31	X-pattern	1.20
AZ31	W-pattern	0.57
PLA/PCL; 70/30 wt.% blend	X-pattern	0.17
PLA/PCL; 70/30 wt.% blend	W-pattern	0.098

## Data Availability

Data are available from the corresponding author on reasonable request.
